# Postcolectomy Peritoneal Environment Increases Colon Cancer Cell Migration Capacity

**DOI:** 10.1155/2016/2540397

**Published:** 2015-12-27

**Authors:** Liron Berkovich, Ronen Ghinea, Salem Majdop, Baruch Shpitz, Ian White, Moshe Mishaeli, Shmuel Avital

**Affiliations:** ^1^Department of Surgery B, Meir Medical Center, 44281 Kfar Saba, Israel; ^2^Sackler Faculty of Medicine, Tel-Aviv University, 6997801 Tel-Aviv, Israel; ^3^Department of Oncology, Meir Medical Center, 44281 Kfar Saba, Israel

## Abstract

*Background.* Clinical data and animal models support an association between postoperative inflammatory response and the risk of colorectal cancer recurrence. Our aim was to evaluate postoperative peritoneal inflammation and its impact on cultured colon cancer cells' migration capacity. *Methods.* 23 patients undergoing elective colorectal resection with uneventful recovery were prospectively enrolled. Patients were operated on for both malignant and benign etiologies. Peritoneal fluids collected at surgery initiation and after surgery were evaluated for their effect on migration potential of human colon cancer cells using an *in vitro* scratch assay and on TNF-*α*, IL-1*β*, IL-6, and IL-10 levels using bead-based fluorokine-linked multianalyte profiling. *Results.* Postoperative peritoneal fluid from all patients increased the migration capacity of colon cancer cells compared to preoperative levels. This effect was significant during the first two postoperative days and decreased thereafter. The increase in colon cancer cell migration capacity correlated with increased levels of peritoneal TNF-*α* and IL-10. *Conclusion.* In this pilot study, we have demonstrated that the intraperitoneal environment following colorectal resection significantly enhances colon cancer cells migration capacity. This effect is associated with postoperative intra-abdominal cytokines level. A larger scale study in colorectal cancer patients is needed in order to correlate these findings with perioperative parameters and clinical outcome.

## 1. Introduction

Curative surgery is the primary treatment for patients diagnosed with colorectal cancer (CRC) and no evidence of metastasis. Within 5 years of surgery, approximately 30% of CRC patients would develop disease recurrence [1].

Clinical studies support the relationship between events that increase perioperative inflammatory response and adverse oncological outcomes in CRC patients. Postoperative infections and anastomotic leaks that modulate the immune system are associated with an increased risk of disease recurrence and decreased disease-free survival [2–4]. Furthermore, studies in animal models have shown that intra-abdominal surgical trauma may increase cancer aggressiveness and the degree of trauma is correlated with the risk of tumor growth and spread [5–7].

Abdominal surgery generates both local and systemic inflammatory responses, characterized by increased systemic levels of stress hormones and local release of cytokines [8, 9]. Postoperative TNF-*α*, IL-6, and IL-10 levels were significantly higher in peritoneal fluids than in peripheral blood, indicating that the peritoneal environment is the source of the corresponding postoperative systemic reaction, specifically in terms of the production of inflammatory mediators [9].

Our basic hypothesis in this study was that the intra-abdominal surgical trauma following colorectal resection for any indication would increase the level of peritoneal inflammatory cytokines coupled with a promalignant effect on cancer cells.

The aim of this study was to evaluate the changes in the effect on cancer cells migration capacity and inflammatory cytokine levels in peritoneal fluids of patients undergoing colorectal resection for both malignant and benign etiologies that had no major complications.

## 2. Methods

This study was registered in the NIH ClinicalTrials.gov Protocol Registration System (Identifiers: NCT02102074, Unique Protocol ID: 0036) and was approved by the IRB Helsinki Committee. The study was performed in accordance with the ethical standards laid down in the Declaration of Helsinki. All patients provided written informed consent before being included in the study.

### 2.1. Patients

A total of 23 patients with malignant or benign disease who were referred for elective surgery were prospectively recruited during the study period. All patients underwent colorectal surgery with an intra-abdominal drain left by the end of the procedure and had no major postoperative complications.

### 2.2. Fluid Sampling

To obtain a baseline, following abdominal access and prior to any surgical step, peritoneal effusion fluids were collected from patients who had over 1 mL volume, which was sufficient for analysis. In patients without sufficient effusion, peritoneal lavage with 100 mL saline was done prior to resection, in accordance with the procedure used by Salvans et al. [4]. Following surgery, 15 mL peritoneal fluid was taken from the patient's abdominal drain 6–10 hours after surgery and daily, for up to 4 days following surgery or until the drain was removed. The samples were centrifuged to discard cells and cell-free fractions were kept frozen at −20°C until experimental use and analysis.

### 2.3. Cell Culture

Human colon cancer cell line SW480 (CCL-228, ATCC, Rockville, MD) was used as a cellular model. Cells were maintained by serial passage in RPMI-1640 medium supplemented with 10% fetal calf sera, 200 *μ*M L-glutamine, 10 units/mL penicillin, and 10 *μ*g/mL streptomycin (Biological Industries, Beit HaEmek, Israel) and kept in a 5% CO_2_ air-humidified atmosphere at 37°C.

### 2.4. Cell Migration Assay

The assay was adopted from the* in vitro* scratch assay protocol [10]. Patient's specimens from all time points were evaluated in a single experiment including a negative control. SW480 cells were cultured in cell culture dishes (6 mm × 15 mm, Corning, NY) and left overnight to form a confluent monolayer. The monolayer was scored to leave a scratch 0.5 mm wide, rinsed with phosphate buffered saline (Biological Industries, Israel), and replaced with fresh, serum-free culture medium containing 20% peritoneal fluid (this concentration was selected as optimal exposure not compromising cell viability, following calibration experiments using 0–100% peritoneal fluids). Negative control of this assay was evaluated using serum-free culture medium with L-glutamine, penicillin, and streptomycin supplementation only. Baseline level of the effect was determined using the presurgical peritoneal effusion or lavage fluids.

Digitized images of the plates were obtained at the start of the experiment (for dish baseline) and after 5 hours (×20, Olympus TH4-200 Microscope). Migration level was determined by the cells counted per scratch area (cells per pixel; Image-Pro Plus 6.0 Software, Media Cybernetics). Bar charts of migration levels were created using Microsoft Office Excel 2007.

### 2.5. Cytokine Evaluation

TNF-*α*, IL-1*β*, IL-6, and IL-10 levels were evaluated in the peritoneal fluids using fluorokine-linked multianalyte profiling assay (bead-based Fluorokine MAP Detection Kit for the Luminex Platform, R&D Systems, Inc.) according to manufacturer's protocol. All cytokines were measured in picograms per milliliter (pg/mL).

### 2.6. Statistical Analysis

Data are presented as numbers for categorical variables and as mean and standard deviation (SD) or median and minimum-maximum for continuous parameters. Migration values were not normally distributed and thus, all group results are presented as median values. Differences between characteristic qualitative variables and levels of migration or cytokines were compared by *t*-test, one-way ANOVA, Mann-Whitney, or Kruskal-Wallis nonparametric tests, each when appropriate. Correlations between two continuous variables were evaluated by Pearson's correlation. Linear regression was used to find predictors of migration. *p* < 0.05 was considered statistically significant. All statistical analyses were performed with SPSS-21 software.

## 3. Results

A total of 23 patients were included in this study. Patient data are presented in Table 1.

### 3.1. The Effect of Peritoneal Fluid on Colon Tumor Cell Migration

The baseline cell migration values evaluated using presurgical peritoneal effusion and lavage fluids were similar (26 × 10^−3^ and 27 × 10^−3^ cells per pixel, resp.). These baseline values were also similar to the assays' negative controls (21 cells per pixel ×10^−3^, *p* = 0.52 and 0.20, resp.). This indicates that the presurgical peritoneal environment did not affect the migration of colon tumor cells in our experimental model.

**Figure 1:**
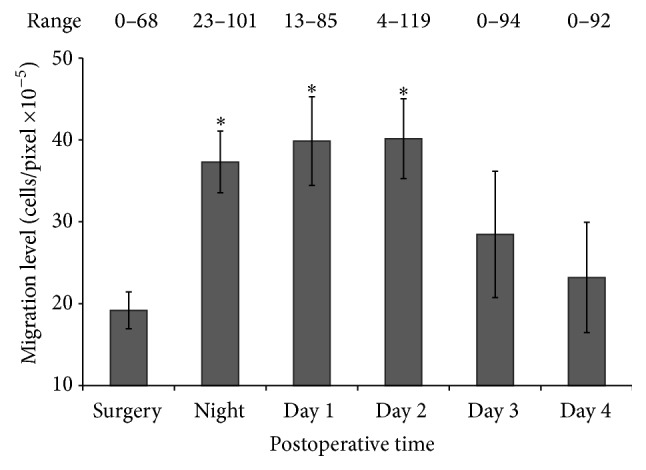
Effect of peritoneal fluids on the migration of colon cancer cells SW480* in vitro*. Median values and standard error bars are displayed. ^*∗*^ *p* < 0.005 relative to the level before surgery.

Universally, in all 23 patients, the cultured colon cancer cells exhibited increased migration with the postoperative peritoneal fluids compared to the preresection fluids (*p* < 0.001). Median migration values were significantly elevated at the postoperative night and at the first and second days postoperatively compared to preresection levels (Figure 1).

Peritoneal fluids obtained on these time points have increased the cell's migration capacity by approximately 2-fold (*p* = 0.0006, *p* = 0.0018, and *p* = 0.0096, resp.). However, migration capacity median values of the third and fourth postoperative days were not significantly elevated when compared to the preresection levels (*p* = 0.0890 and *p* = 0.1659, resp.).

**Figure 2:**
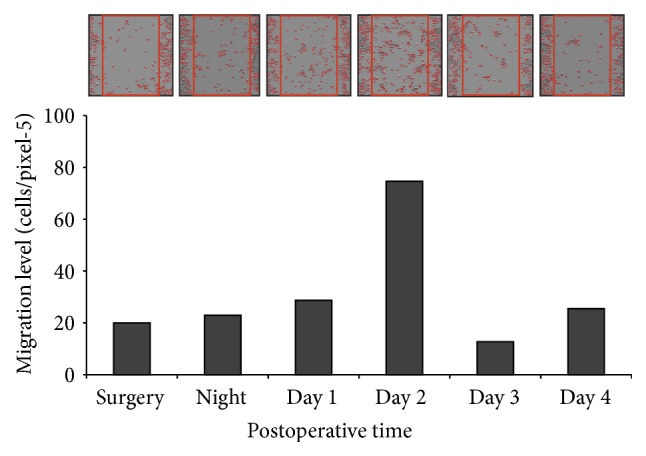
The effect of postoperative peritoneal fluids from one individual patient on the migration capacity of colon cancer cells SW480* in vitro*. The correlated microscopic images are shown above the bars.

An example for the effect of postoperative peritoneal fluids from one individual patient on the cell's migration capacity is illustrated in Figure 2.

No clinical or operative characteristics such as age or etiology were associated with the level of cell migration capacity in a multivariate analysis.

### 3.2. Peritoneal Cytokine Levels and Association with Migration Capacity

**Figure 3:**
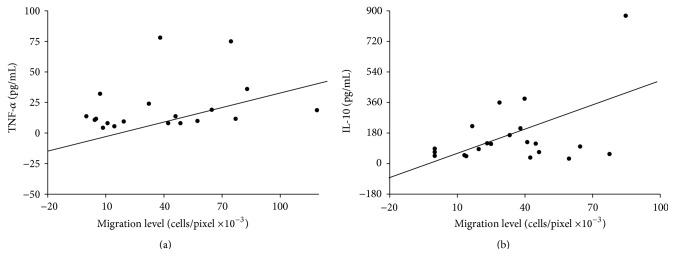
Pearson correlation analysis of postoperative induction of colon cancer cell migration and TNF-*α* and IL-10 levels. (a) The effect of peritoneal fluid on the migration capacity correlated with its TNF-*α* level on the second postoperative day. Pearson *r* = 0.507; *p* = 0.032. (b) The effect of peritoneal fluid on the migration capacity correlated with its IL-10 levels on the first postoperative day. Pearson *r* = 0.537; *p* = 0.018.

Significant postoperative elevations were found in peritoneal cytokines TNF-*α*, IL-1*β*, IL-6, and IL-10, relative to presurgical levels (*p* < 0.05, Table 2). Increased migration was significantly correlated with IL-10 levels on the first postoperative day (Pearson's *r* = 0.537, *p* = 0.018) and with TNF-*α* levels on the second postoperative day (Pearson's *r* = 0.507, *p* = 0.032) (Figure 3). Although not statistically significant, the effect was also noted in other cytokines. No such correlation was found with peritoneal IL-1*β* and IL-6.

## 4. Discussion

In this study we have evaluated the effect of peritoneal fluids following uneventful colorectal surgery on the migration capacity of colon cancer cells in the first postoperative days. We have found that in all patients, regardless of surgical etiology, a significant increase in migration capacity was evident in the first two postoperative days. Colon cancer migration capacity was partly correlated with TNF-*α* and IL-10.

This was a pilot study with heterogeneous group of patients and thus was underpowered to look for correlation between migration capacity and perioperative parameters. However, it clearly demonstrated a phenomenon of increased migration capacity of colon cancer cells in the early postoperative days.

The hypothesis by which early postoperative processes may augment the spread and outgrowth of CRC metastases is based on several features. Disseminated colon tumor cells are evident in CRC patients and their spread to the circulation or the peritoneal cavity may increase during surgery [11], constituting potential secondary metastasis. Additionally, within hours after surgery, a transient suppression of immunologic functions occurs [12, 13], accompanied by a boost in secretion of proinflammatory mediators such as hormones and cytokines [14]. These circumstances support tumor cell viability and adherence to endothelial cells and, thus, the ability to metastasize [15, 16]. As disseminated tumor cells have a limited life span [17], these dynamics peak during surgery and the early postoperative period and may have significant clinical impacts. Therefore, the perioperative period has emerged as a significant factor in determining recurrence [5, 7].

This hypothesis has been supported by clinical studies using* in vitro* experimental models. Kirman et al. [18] demonstrated that postoperative plasma of patients undergoing colon resection increased the mitogenic activity of cultured colon cancer cells compared to preoperative plasma. This effect correlated with the length of the surgical incision, indicating an association of this effect with the operative trauma.

A recent study by Salvans et al. [4] demonstrated that peritoneal fluids and serum obtained 4 days after surgery from CRC patients who had peritoneal infections enhanced the migration of cultured colon cancer cells compared to patients who did not experience an infection. Furthermore, enhanced migration capacity in these patients was associated with early cancer recurrence. In that study, the peritoneal fluids elevated the migration capacity compared to serum samples.

As was previously noted, in case of abdominal surgery, the postoperative peritoneal environment is the source of the corresponding systemic inflammatory reaction, characterized by different homeostasis than the peripheral blood [9]. Therefore, the effect of the postoperative peritoneal environment on tumor cells may be represented* in vitro* more precisely than in the peripheral environment.

Our study evaluated patients who had uncomplicated abdominal surgery in order to explore the normal physiological effect of colectomy on colon cancer cells. We found that the peritoneal fluid from all patients, with either cancer or benign colon diseases, increased the migration capacity of colon cancer cells postoperatively. Overall, this effect was significant during the first two postoperative days and declined by the third day. Based on these results, we conclude that the promigration effect of peritoneal fluid is associated with the magnitude of the peritoneal inflammatory response and cytokine secretion.

It has been shown that the inflammatory response to surgical trauma presented by cytokines and acute phase reactants peak during surgery and the first postoperative day and then gradually decline [19, 20]. Thus, we think that the decrease in the migration capacity observed on postoperative day 3 correlates with the decrease in the inflammatory physiological response.

The role of TNF-*α* in promoting colon cancer metastasis was demonstrated via an experimental murine cancer metastasis model in which a colon adenocarcinoma cell line generated lung metastases [21]. In this model, metastatic growth response depended on TNF-*α* production by host hematopoietic cells. Another study showed that TNF-receptor deficient mice are resistant to the development of liver metastases in an experimental colon cancer model [22]. However, additional research is needed to determine the clinical cause and effects of TNF-*α* and specific mediators in this process. This can be done also by including specific cytokines inhibitors in our* in vitro* experimental model.

In this study we used the scratch assay [10] to evaluate colon cancer migration capacity. This assay is a well documented assay; however, a further evaluation with invasion assays may add to the validity of our results.

While the increased inflammatory response and increased migration capacity of colon cancer cells following colorectal surgery were evident in every patient, multiple variables may affect each patient's specific level of reaction. Clinical parameters related to the patient (age, comorbidities, medications, etiology, etc.) and to the surgery (operative time, extent of colonic resection, surgical approach, etc.) are potential factors that could affect the postoperative inflammatory response and colon cancer cells migration capacity levels. However, a major limitation of our study is the small sample size of patients that prevent a statistical analysis to evaluate the impact of these specific potential factors. Additionally, the clinical significance of our findings should be further evaluated in a larger number of patients with long term follow-up.

However, a clinical implication of our findings may suggest a perioperative use of anti-inflammatory drugs in patients undergoing colorectal resection to reduce the inflammatory response and the consequential effect on tumor cells migration capacity.

Nonsteroidal anti-inflammatory drugs (NSAIDs) are the most common and available anti-inflammatory drugs and might be considered for this purpose. However, accumulated data reveal that NSAIDs are associated with increased risk of anastomotic leaks following colorectal surgery [23, 24].

We believe that the role of other specific anti-inflammatory drugs should be explored. As our data indicates an association between TNF-*α* and colon cancer migration capacity it would be reasonable to evaluate a drug such as Infliximab (monoclonal anti TNF-*α* antibody) which has no association with postoperative adverse side effects [25].

In conclusion, the intraperitoneal environment following colorectal resection enhances colon cancer migration capacity in patients with no postoperative major complications. This effect is significant during the first two postoperative days and is linked to the postoperative intra-abdominal inflammatory response. Further research is needed to fully elucidate the role of specific mediators such as TNF-*α* in this process and to evaluate whether reducing the postoperative inflammatory response will reduce migration potential of cancer cells.

## Figures and Tables

**Table 1:** Patients' clinical characteristics and operative data.

Parameter	Value
	(*N* = 23)
Age, years, mean ± SD	55.5 ± 16.2
BMI, mean ± SD	26.1 ± 6.4
Male/female	6/17
Etiology	
Benign condition (IBD, diverticular disease, polyps)	15
Colorectal adenocarcinoma	8
Procedure	
Total colectomy	2
Right sided colectomy	4
Left sided colectomy	7
Proctectomy	10
Laparoscopy, open	15, 8
Operative time, hr, median (range)	4:38 (1:49–10:17)

**Table 2:** Cytokine levels in peritoneal fluids of patients during and after surgery.

Cytokine levels (pg/mL)	Surgery	6–10 hours after surgery	Day 1	Day 2	Day 3	Day 4
TNF-*α*	5.4	14.0	14.0	11.6^*∗*^	14.1^*∗*^	19.0^*∗*^
	(4.0–20.0)	(2.5–54.3)	(2.5–295.0)	(4.3–78.0)	(3–284.6)	(1.9–76.0)

IL-1*β*	1.9	34.7^*∗*^	30.1^*∗*^	22.1^*∗*^	21.1^*∗*^	27.5^*∗*^
	(0.5–2.0)	(6.6–826.2)	(1.9–453.5)	(3.0–142.2)	(1.3–393.5)	(2.0–815.8)

IL-6	87	8584^*∗*^	8463^*∗*^	6570^*∗*^	5867^*∗*^	3853^*∗*^
	(2–2236)	(4562–13504)	(5176–13563)	(2683–11198)	(797–9862)	(299–245531)

IL-10	3.1	133.7^*∗*^	108.2^*∗*^	23.7	18.1	17.9
	(0.0–351.9)	(3.3–32722.0)	(29.1–7813.7)	(2.9–263.5)	(0.5–1191.5)	(0.4–145.0)

Median and range values are displayed; ^*∗*^ *p* < 0.05 relative to levels before surgery.
